# Local adaptation does not lead to genome‐wide differentiation in lava flow lizards

**DOI:** 10.1002/ece3.5231

**Published:** 2019-05-29

**Authors:** Alexander R. Krohn, Eveline T. Diepeveen, Ke Bi, Erica Bree Rosenblum

**Affiliations:** ^1^ Department of Environmental Science, Policy and Management University of California, Berkeley Berkeley California; ^2^ Museum of Vertebrate Zoology University of California, Berkeley Berkeley California; ^3^ Department of Bionanoscience, Kavli Institute of NanoScience, Faculty of Applied Sciences Delft University of Technology Delft The Netherlands; ^4^ Computational Genomics Resource Laboratory (CGRL), California Institute for Quantitative Biosciences (QB3) University of California, Berkeley Berkeley California

**Keywords:** adaptation, isolation by environment, lava flow, lizards, RADseq

## Abstract

Adaptation can occur with or without genome‐wide differentiation. If adaptive loci are linked to traits involved in reproductive isolation, genome‐wide divergence is likely, and speciation is possible. However, adaptation can also lead to phenotypic differentiation without genome‐wide divergence if levels of ongoing gene flow are high. Here, we use the replicated occurrence of melanism in lava flow lizards to assess the relationship between local adaptation and genome‐wide differentiation. We compare patterns of phenotypic and genomic divergence among lava flow and nonlava populations for three lizard species and three lava flows in the Chihuahuan Desert. We find that local phenotypic adaptation (melanism) is not typically accompanied by genome‐wide differentiation. Specifically, lava populations do not generally exhibit greater divergence from nonlava populations than expected by geography alone, regardless of whether the lava formation is 5,000 or 760,000 years old. We also infer that gene flow between lava and nonlava populations is ongoing in all lava populations surveyed. Recent work in the isolation by environment and ecological speciation literature suggests that environmentally driven genome‐wide differentiation is common in nature. However, local adaptation may often simply be local adaptation rather than an early stage of ecological speciation.

## 1 INTRODUCTION

Given that environmental differences can lead to genetic differentiation among populations—and even speciation—it is crucial to understand the effects of geography and ecology on genomic differentiation. There are a number of ways that geographic and ecological factors can lead to genome‐wide differentiation across populations. A common pattern is isolation by distance (IBD) (Wright, 1943), whereby gene flow across populations decreases with physical distance. Environmental differences can also lead to genomic differentiation—independent of geographic distance—referred to as isolation by environment (IBE) (Sexton, Hangartner, & Hoffmann, 2014; Wang & Bradburd, 2014). Environmental transitions can generate genomic divergence with or without adaptation, for example, when extrinsic barriers reduce gene flow across an ecotone. Isolation by adaptation (IBA) (Nosil, Egan, & Funk, 2008) is a particular case of IBE, in which adaptation plays a central role in genomic patterns of divergence. Cases of IBA demonstrate that adaptation can lead to genome‐wide differentiation due to linkage among loci and/or restricted gene flow promoting genome‐wide genetic drift (Funk, Egan, & Nosil, 2011; Nosil et al., 2008).

Of course, adaptation does not always lead to genome‐wide differentiation, especially when locally adaptive phenotypes have a simple genetic basis and no consequences for reproductive isolation. While examples of IBA and IBE are increasingly common in the literature (Funk et al., 2011; Orsini, Vanoverbeke, Swillen, Mergeay, & Meester, 2013; Seehausen et al., 2008; Sexton et al., 2014; Shafer & Wolf, 2013), some authors suggest that there may be a publication bias against studies that do not find a correlation between genetic differentiation and environmental differences (Feder, Egan, & Nosil, 2012; Shafer & Wolf, 2013). Thus, it is possible that cases where patterns of genomic variation across environmental gradients do not follow expectations of IBE or IBA are underreported in the literature. Thus, replicated studies across species and evolutionary timescales are needed to understand the frequency with which—and the conditions under which—local adaptation does or does not lead to genome‐wide differentiation in the wild.

The lava flows of the Chihuahuan Desert in New Mexico represent an ideal place to study whether patterns of IBA and IBE are shared across evolutionary and environmental replicates because multiple species are found on multiple independent lava flows. The dark basalt rocks of the lava flows contrast with the surrounding adobe colored soils, and many vertebrates exhibit darker—melanistic (Majerus, 1998)—color morphs on the lava flows (Best, James, & Best, 1983; Hoekstra & Nachman, 2003; Lewis, 1949, 1951; Rosenblum, 2005). Given that numerous lava flows are within approximately 100 miles of each other in the Chihuahuan Desert, they draw from a similar pool of colonists and represent multiple replicates of colonization, adaptation, and community assembly. The lava flows vary in age and substrate color, representing snapshots of evolution since initial colonization. The youngest lava flow is only 5,000 years old and has more homogeneously black substrate (Dunbar, 1999). The more ancient lava flows are orders of magnitude older (106,000 and 760,00 years old) and have more sand accumulation creating a more heterogeneously colored background (Bachman & Mehnert, 1978; Hoffer, 1975).

A number of reptile species have been reported to be significantly darker on lava flows in the Chihuahuan Desert than in the surrounding desert (Krohn & Rosenblum, 2016; Lewis, 1951; Rosenblum, 2005). Melanistic phenotypes in lava flow lizards are typically considered to be an adaptation for crypsis because visually hunting diurnal predators are known to prey on color‐mismatched lizards (Luke, 1989). Melanistic lava flow lizards of the focal species typically match their background extremely well, often better than lizards from surrounding nonlava flow populations (Krohn & Rosenblum, 2016; Rosenblum, 2005). Dark coloration in lava flow lizards also appears to be heritable as the degree of melanism does not change ontogenetically, is not influenced by the juvenile's substrate color, and correlates strongly with parental coloration (Corl, Lancaster, & Sinervo, 2012; Micheletti, Parra, & Routman, 2012; Rosenblum, 2005). Additionally, while lizards can change color over short time periods, physiological color change is insufficient to explain the observed differences in coloration in these species (Krohn & Rosenblum, 2016; Micheletti et al., 2012; Rosenblum, 2005).

Although melanism appears to be heritable and associated with environmental variation in many reptile species, it is not clear whether local adaptation in coloration is expected to lead to genome‐wide differentiation. On one hand, patterns may simply reflect migration–selection balance, where selection on relatively few genes is balanced by ongoing gene flow and genome‐wide divergence is unlikely (Corl et al., 2018; Rosenblum, Hickerson, & Moritz, 2007). On the other hand, color variation in reptiles can have consequences for both natural and sexual selection as many lizard species use visual cues including coloration for mate choice (Hardwick, Robertson, & Rosenblum, 2013; Robertson & Rosenblum, 2009). In this case, local adaptation could lead to genome‐wide divergence and ultimately reproductive isolation. Thus, empirical tests are necessary to determine whether local adaptation typically leads to genomic differentiation between lava flow populations and nonlava flow lizards.

Here, we focus on understanding patterns of local adaptation and genomic differentiation in three lizard species distributed across three lava flows in the Chihuahuan Desert. The three focal species—*Crotaphytus collaris, Sceloporus cowlesi,* and *Urosaurus ornatus*—have all been reported to have melanic populations on local lava flows (Krohn & Rosenblum, 2016; Lewis, 1951; Rosenblum, 2005). The three lava flows differ in age—from 5,000 to 106,000 to 760,000 years old—providing an opportunity to evaluate whether divergence accumulates (or decays) over time. For populations on and off the lava flows, we quantified color variation, measured genetic variation using restriction‐associated DNA sequencing (Peterson, Weber, Kay, Fisher, & Hoekstra, 2012), and estimated migration rates using demographic simulations. Our sampling approach leveraged population trios (where we compared each focal lava flow population with two nearby nonlava flow populations) to disentangle the effects of IBD and IBE.

## 2 MATERIALS AND METHODS

### 2.1 Phenotypic data

#### 2.1.1 Sampling and color quantification

**Figure 1:**
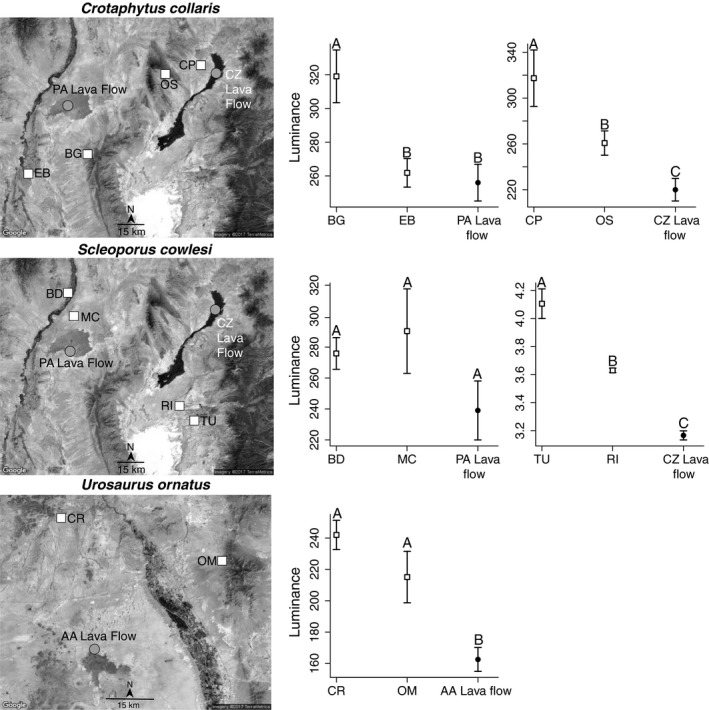
Sampling locations and phenotypic variation in lizards on and off focal lava flows. Squares represent nonlava flow populations, while circles represent lava flow populations. Each row represents a different focal species. In each phenotype panel, the mean and standard error of luminance are shown. Different letters above the error bars correspond to significantly different groupings of populations according to post hoc Tukey's tests. Satellite images courtesy of Google Earth and Digital Globe

We sampled five populations of three species of lizard across three lava flows (Figure 1). We caught lizards from the Carrizozo Lava Flow (CZLF; ~5,200 years old (Dunbar, 1999)), Aden Afton Lava Flow (~106,000 years old, (Hoffer, 1975)), and Pedro Armendariz Lava Flow (PALF; ~760,000 years old (Bachman & Mehnert, 1978)) in south‐central New Mexico. Our sampling scheme focused on population trios: for each lava flow lizard population, we also caught lizards from two nonlava flow populations (Figure 1). The nonlava flow populations were as geographically close to the lava flows as possible. For clarity, each population trio will be referred to by an abbreviated version of the lava flow name and a species. All sampling occurred between June and August, 2013 and 2015.

At CZLF, we quantified the dorsal coloration of 18 *C. collaris* (CZLF‐*Crotaphytus*) and 26 *S. cowlesi* (CZLF‐*Sceloporus*). We included additional phenotypic data of *S. cowlesi* at CZLF from a previous study (Rosenblum, 2006). Surrounding the CZLF (nonlava flow populations), we quantified the dorsal coloration of five *C. collaris* from Carrizozo Private Land Partnership (CP), 18 *C. collaris* from the Oscura Mountains (OS), seven *S. cowlesi* from Tularosa (TU), and six *S. cowlesi* from Rita Site (RI). At Aden Afton Lava Flow (AALF), we quantified the dorsal coloration of eight *U. ornatus* (AALF‐*Urosaurus*). Surrounding the Aden Afton Lava Flow, we quantified the dorsal coloration of seven *U. ornatus* from Corralitos Ranch Road (CR) and six from the Organ Mountains (OM). At the PALF, we quantified dorsal coloration in 14 *C. collaris* (PALF‐*Crotaphytus*) and five *S. cowlesi* (PALF‐*Sceloporus*). Surrounding the PALF, we quantified the dorsal coloration of 23 *C. collaris* from Elephant Butte (EB), five *C. collaris* from Big Gyp Mountain (BG), five *S. cowlesi* from Mesa Camp (MC), and five *S. cowlesi* from Bosque del Apache (BD; Table S1).

For all populations’ trios except the CZLF‐*Sceloporus* population trio, we used photographs to quantify dorsal coloration. We followed the procedures outlined in Krohn and Rosenblum (2016) to take standardized photographs of the lizards, using the same camera and camera settings as in Krohn and Rosenblum (2016). Briefly, we photographed lizards at their mean operating body temperature (*C. collaris* at 36°C, *S. cowlesi* at 35°C and *U. ornatus* at 35°C) according to Brattstrom (1965). We photographed all lizards on white paper in an upside‐down bucket in direct sunlight to allow for a diffuse, constant light source for all photographs. All photographs included a white, gray, and black standard (Adorama QPcard 101) for color standardization. We imported photographs in RAW format, linearized the photographs using the gray and white standards in Photoshop, and then exported the photographs in the lossless TIF format (Krohn & Rosenblum, 2016). To quantify overall dorsal coloration, we took the average *R*, *G*, and *B* values across a 75 × 75 pixel square in *C. collaris (*Krohn & Rosenblum, 2016), across the dorsal midline stripe in *S. cowlesi *(Rosenblum, 2006), and across the entire dorsal body surface of *U. ornatus* in ImageJ using the plugin RGB measure (v1.47; National Institutes of Health). To convert the RGB values to a color space meaningful for most vertebrate visual systems, we used properties of color and brightness opponency (Endler, 2012; Endler & Mielke, 2005; Osorio, Vorobyev, & Jones, 1999) to calculate values of hue, chroma, and luminance from the RGB values as in Krohn and Rosenblum (2016). Given that luminance explains significant differences in coloration between lava flow and nonlava flow lizards (Krohn & Rosenblum, 2016; Rosenblum, 2006), we used luminance to compare lizard coloration in all our subsequent analyses.

We quantified coloration of the CZLF‐*Sceloporus* population trio using data collected for a prior study using a spectrophotometer. For CZLF‐*Sceloporus* and RI, we integrated the raw reflectance data from Rosenblum (2006). Because lizards from TU were collected after the Rosenblum (2006) study, we collected and analyzed reflectance data identically, but used an Ocean Optics Jaz spectrophotometer. As above, and in Rosenblum (2006), we compared the luminance of *S. cowlesi* at active body temperature.

For each trio of populations, we used an ANOVA to determine whether populations varied significantly in luminance. We ran an ANOVA with luminance values as the dependent variable and population as the independent variable. If a result was significant, we ran post hoc Tukey's tests to determine which populations differed significantly in luminance. We used R (v. 3.3.1, R Development Core Team, 2008) for all our statistical analyses. The University of California, Berkeley, Institutional Animal Care and Use Committee approved all animal handling, color measurement, and tissue sampling (protocol R347‐0314).

### 2.2 Genotypic data

#### 2.2.1 Sampling and RAD sequencing

We used a subset of the same individuals used in the phenotypic analyses for the genotypic analyses. Given that accurate inferences can be made from few individuals per population when one uses individual‐based population genetics methods and samples thousands of loci per individual (Barley, Monnahan, Thomson, Grismer, & Brown, 2015; Felsenstein, 2006; Prunier et al., 2013), we sequenced 5–8 individuals from each lava flow population, and each of the two nonlava flow populations. We constructed a double‐digest RADseq library (Peterson et al., 2012), for 96 individuals of our three focal species (*n* = 46 *C. collaris* total; *n* = 35 *S. cowlesi* total; *n* = 15 *U. ornatus* total) using the enzymes SphI and MluCI. We followed Peterson et al. (2012) except that we used PippinPrep (Sage Science) for size selection instead of beads. We size‐selected fragments between 420 and 620 bp in length and ran a final PCR with 12 cycles. We sequenced the library twice on one lane of the Illumina HiSeq 2500 at the Vincent J. Coates Genomic Sequencing Laboratory at UC Berkeley with 100 bp single‐end reads.

#### 2.2.2 RAD sequence processing and filtering

Before analyzing our data, we filtered our reads to ensure only high‐quality loci. We followed the RADToolKit pipeline (https://github.com/CGRL-QB3-UCBerkeley/RAD) to demultiplex individuals, remove low‐quality reads, create individual contigs, and to create a de novo reference assembly. We demultiplexed individuals while allowing for one mismatch per barcode. After demultiplexing, we separated individuals by species and continued the pipeline on each species independently. We filtered out reads that were low quality (Phred score <20), were contaminated by bacteria, had anomalous runs of a single base (more than 50% of the read), or did not contain the restriction enzyme cut site (allowing a mismatch of 1 bp). After filtering, we removed individuals that did not have enough reads to continue (fewer than 250,000 reads per individual; *n* = 14 *C. collaris*, *n* = 9 *S. cowlesi*, *n* = 2 *U. ornatus*). Next, we masked repetitive elements known from vertebrate metazoans using RepeatMasker (version 4.0, 2013–2015) and clustered reads into contigs for each individual using CD‐hit (Fu, Niu, Zhu, Wu, & Li, 2012; Li & Godzik, 2006), with a sequence identity threshold between 0.8 and 0.95 (optimized for each species using the program “cluster test” from RADToolKit). We only kept clusters with at least two reads and discarded loci if the percentage of uncalled bases exceeded 80%. We then combined the resulting RAD contigs from each individual using CD‐hit with a sequence identity threshold of 0.8. We created a reference genome for the species by including shared contig sets present in at least 70% of the individuals. We aligned cleaned reads of each individual to the reference genome using Novoalign (v. 3.02.12; Novocraft Technologies, Selangor, Malaysia) and only mapped read groups that mapped uniquely to the reference genome. We used Picard (http://picard.sourceforge.net) to add read groups to the alignment and GATK (McKenna et al., 2010) to realign reads around indels. We only kept sites that were biallelic, not within 5 bp of an indel, had low mapping or base calling scores, or that had excessive heterozygosity (to purge potential paralogs; one‐tailed HWE test, *p* < 1 × 10^−4^).

For each species, we tried to maximize the number of high‐quality loci, while minimizing the amount of missing data. For *C. collaris*, we kept loci only if they were present in 80% of individuals with at least 8X coverage. For *S. cowlesi*, we only kept loci if they were present in 70% of individuals at 5X coverage. For *U. ornatus,* we kept loci only if they were present in 70% of individuals with at least 5X coverage. For subsequent analyses, we used Analysis of Next Generation Sequence Data (ANGSD; Korneliussen, Albrechtsen, & Nielsen, 2014) on the filtered set of sites. ANGSD incorporates statistical uncertainty associated with genotype calls in the form of site allele frequency likelihoods, genotype likelihoods, or genotype posterior probabilities into its analysis. Thus, because it incorporates uncertainty into the analyses, ANGSD is favorable for low to medium coverage datasets. Wherever possible, we performed analyses using genotype likelihoods and/or posterior probabilities. When we did have to call SNPs, we only included highest quality sites (see below for the specifics of each analysis).

### 2.3 Data analysis

First, to understand basic population structure, we visualized genetic data for each species using a principal components analysis (PCA). We used the program ngsCovar to create a covariance matrix for each individual's genotypes (Fumagalli et al.., 2013). ngsCovar accounts for the uncertainty of each genotype by incorporating genotype posterior probabilities to generate the covariance matrix. For each trio of populations, we input genotype posterior probabilities from ANGSD. We called genotype posterior probabilities (not including genotypes with a posterior <0.95) at sites that, in addition to being present after the filtering described above, had a minimum coverage of 5X, were polymorphic (using a likelihood ratio test with a minimum *p*‐value of 1 × 10^−2^), had a minor allele frequency >0.05, and were present in at least 80% of individuals. We plotted the eigenvalues of the covariance matrix in R to visualize population structure in each of the population trios.

Second, to understand the relationship between genetic variation and geographic distances, we calculated a pairwise genetic distance matrix for all individuals in each of the population trios. We used ngsDist to calculate genetic distance between all individuals (Vieira, Lassalle, Korneliussen, & Fumagalli, 2016). ngsDist takes genotype uncertainty into consideration by calculating genetic distances from genotype likelihoods or posterior probabilities instead of directly from the genotype calls. Using the same filters as above, we input genotype posterior probabilities from ANGSD into ngsDist to generate a genetic distance matrix for the individuals of each population trio. To determine whether genetic distance increases more between lava and nonlava flow comparisons of individuals than one would expect given their geographic distance, we plotted pairwise genetic distance with pairwise geographic distance and colored each pairwise comparisons by whether it was between two individuals from different environments (i.e., lava flow and nonlava flow individuals) or the same environment (i.e., both from a lava flow or both not from a lava flow).

Third, we quantified the role of geographic distance and ecological distance in shaping the observed genetic distances using Mantel tests. For each population trio, we called SNPs using ANGSD. In addition to the filters applied in the RAD Sequence Processing and Filtering section, we further filtered sites to those that had a minimum coverage of 5X, were polymorphic (using a likelihood ratio test with a minimum *p*‐value of 1 × 10^−6^), had a minor allele frequency >0.05, and had a genotype posterior probability cutoff of at least 0.95. We calculated pairwise distances from field‐collected longitude and latitude data using the R package raster (Hijmans & van Etten, 2012). For our ecological distance matrix, we scored comparisons between individuals on different habitats (i.e., lava flow to nonlava flow comparisons) as 1 and comparisons between the same habitats (i.e., lava flow to lava flow or nonlava flow to nonlava flow) as 0. For each population trio, we ran one Mantel test and one partial Mantel test. First, to determine whether genetic distance increases with geographic distance as predicted under IBD (Wright, 1943), we ran a Mantel test with genetic distance as the dependent variable and geographic distance as the independent variable. Second, to determine if genetic distance increases with environmental distance while controlling for the effect of geographic distance, we ran a partial Mantel test with genetic distance as the dependent variable, ecological distance as the independent variable, and geographic distance as the variable being held constant. We used the vegan package (Oksanen et al., 2017) in R for all Mantel tests. Given that partial Mantel tests have particularly high false‐positive rates when geographic and environmental distances are spatially autocorrelated (Bradburd, Ralph, & Coop, 2013; Guillot & Rousset, 2013), we also calculated levels of spatial autocorrelation for each population trio using Moran's I in the R package ape (Paradis, Claude, & Strimmer, 2004).

Finally, to determine whether gene flow might contribute to observed patterns of genetic differentiation between lava flow and nonlava flow populations, we calculated *F*_ST_ and used diffusion approximation models to approximate migration rates. We used ANGSD and its built‐in function realSFS to calculate *F*_ST_. For each population trio, we calculated *F*_ST_ based on the site frequency spectrum of every pairwise combination of populations (three combinations per population trio). *F*_ST_ values can reflect processes of divergence, processes of migration, or combinations therein (Nielsen & Slatkin, 2013), so more explicit analyses are also needed to quantify migration. We thus used *∂a∂i* (Gutenkunst, Hernandez, Williamson, & Bustamante, 2009) to more explicitly quantify rates of migration onto and off of each lava flow in a population trio using diffusion approximation models. Within each population trio, we approximated models of migration for each pairwise combination of populations. Thus, each trio had the same three two‐population comparisons as in the *F*_ST_ analyses above, for a total of 12 comparisons. For each comparison of two populations, we called SNPs identically as in our Mantel analyses, but using –doGeno 2 in ANGSD to output allele calls. We converted the allele calls into the *∂a∂i* input format using custom Python scripts (available at https://github.com/alexkrohn/LavaFlowLizards/). Using *∂a∂i*, we created joint site frequency spectra for each pair of populations. We ran four models of demographic scenarios to model how alleles might change with the formation of the lava flow and ongoing migration. We ran a neutral model with no divergence, a model with divergence but no migration, a model with divergence and symmetric present‐day migration, and a model with divergence and asymmetric present‐day migration (as in Portik et al., 2017). For each population comparison and each model, we followed the procedure of Portik et al. (2017) to optimize parameters. We optimized our original parameters over three rounds. To get the best final estimates of our parameters, each round of optimization had an increasing amount of iterations of parameter optimization, an increasing amount of total replicates from modified starting parameters, and a decreasing degree to which parameters were perturbed (Portik et al., 2017). From each round, we took the parameters with the lowest AIC score to continue to the next round of optimization (Ese et al., 2016). The first round had 20 iterations over 50 replicates, the second round had 30 iterations over 50 replicates, and the final round had 50 iterations over 100 replicates. We determined that a model best described the data when its AIC score was at least 10 less than the next best model (Ese et al., 2016). Scripts of models used and optimization protocols are available online (https://github.com/dportik/dadi_pipeline/tree/master/Two_Populations).

## 3 RESULTS

Although we observed strong geographic color variation among the populations sampled, only certain lava flow populations were melanistic. Luminance varied significantly among populations in all trios except the trio that included PALF‐*Sceloporus* (values shown for trio comparisons that include the indicated lava flow population; CZLF‐*Crotaphytus*: *F* = 10.19, *df* = 2, *p < *0.001; PALF‐*Crotaphtyus*: *F* = 5.05, *df* = 2, *p* = 0.011; CZLF‐*Sceloporus*: *F* = 81.2, *df* = 2, *p < *0.001; AALF‐*Urosaurus*: *F* = 14.77, *df* = 2, *p* < 0.001; PALF‐*Sceloporus*: *F* = 1.86, *df* = 2, *p* = 0.197). Specifically, only CZLF‐*Crotaphytus* and CZLF‐*Sceloporus* (all post hoc Tukey's test *p*‐values < 0.02; Figure 1), and AALF‐*Urosaurus* (all post hoc Tukey's test *p‐*values < 0.01; Figure 1) were significantly darker than the surrounding nonlava populations of each species. Thus, only populations of lizards on the Carrizozo and Aden Afton Lava Flows, but not the PALF are melanistic according to this analysis (as defined by Majerus, 1998).

For our genomic analyses, we recovered 262,338,151 reads from two runs of sequencing. After filtering reads for quality and removing individuals with too much missing data, we recovered different numbers of sites and contigs per species. For *C. collaris*, we recovered 1,151,763 high‐quality sites on 15,369 contigs with a median coverage of 25.5X (range: 5.1X–92.7X) from 28 individuals. For *S. cowlesi*, sequences from CZLF‐*Sceloporus* fell below our quality thresholds and thus were excluded from further analysis. Using only individuals from the PALF‐*Sceloporus* population trio, we assembled a new de novo reference assembly and aligned reads for analysis as above. We recovered 748,818 high‐quality sites on 10,426 contigs with a median coverage of 14.85X (range: 6.7X–141.1X) for those 12 *S. cowlesi*. For *U. ornatus*, we recovered 1,460,625 high‐quality sites on 20,051 contigs with a median coverage of 20.5X (range: 5.8X–45.9X) from 13 individuals. Raw data are archived on the Short Read Archive (BioProject Accession PRJNA417310), and scripts to reproduce this workflow are available at https://github.com/alexkrohn/LavaFlowLizards.

**Figure 2:**
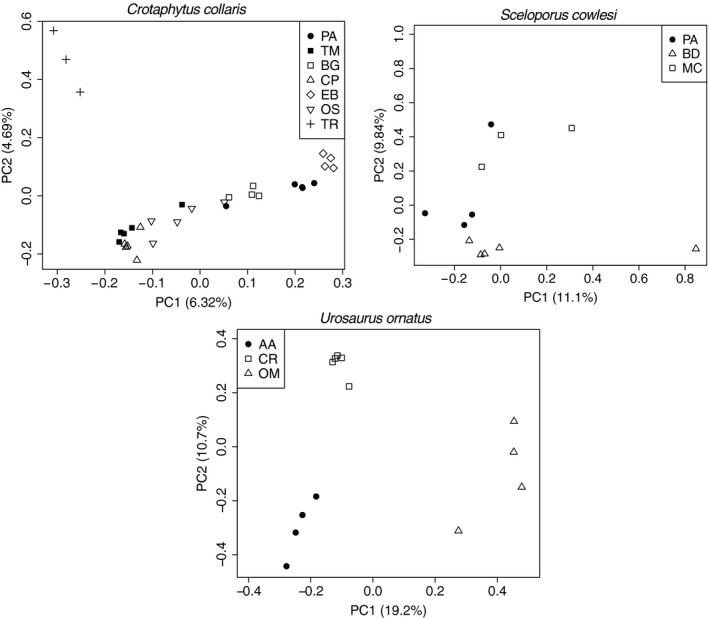
Principle component analyses (PCA) of genetic variation in lava flow and nonlava flow lizards. Filled shapes represent individuals sampled from lava flows, and open shapes represent individuals sampled from nonlava populations. Population locations—with identical abbreviations—are shown in Figure 1. While there is at least some population‐level clustering in all three species, lava populations are not more distinct than expected based on background divergence. Figure 2 and Table 1 explicitly test the contribution of isolation by distance and isolation by environment to observed patterns

**Figure 3:**
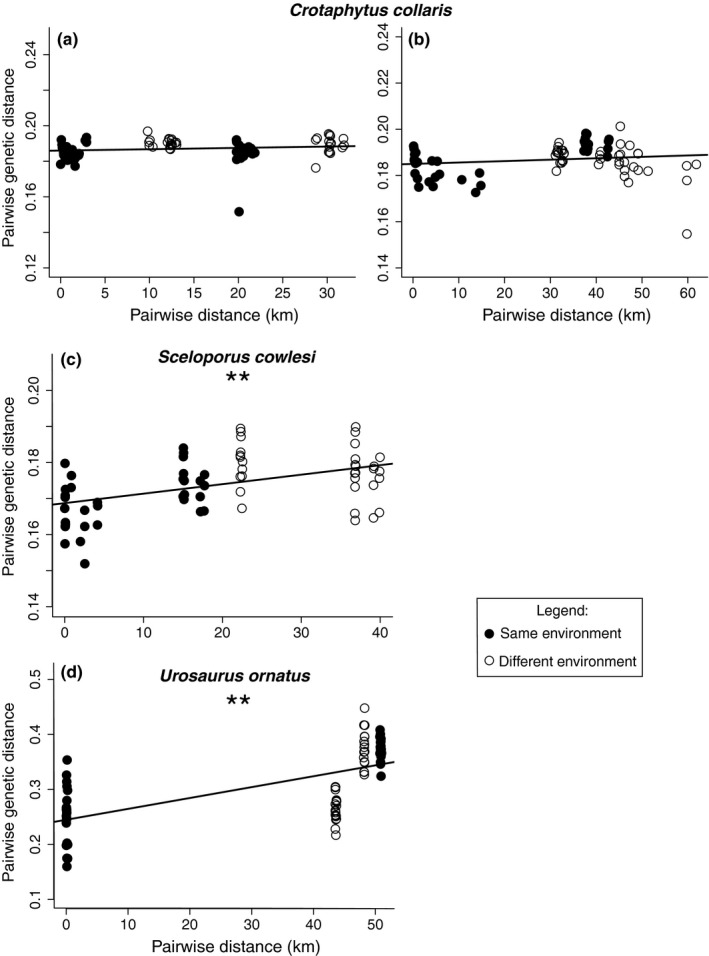
Isolation by environment plots in three species of lava flow lizards. Panels (a and b) are data from populations of *Crotaphytus collaris* on and around the Carrizozo Lava Flow and Pedro Armendariz Lava Flow, respectively. Panel (c) is from populations of *Sceloporus cowlesi* on and around the Carrizozo Lava Flow. Panel (d) is from populations of *Urosaurus ornatus* on and around the Aden Afton Lava Flow. Each point is a pairwise comparison of two individuals, colored by the type of comparison (i.e., lava flow to lava flow, or nonlava flow to nonlava flow = same environment; nonlava flow to lava flow = different environment). Trend lines indicate the regression of pairwise genetic distance against pairwise geographic distance. Pairwise genetic distance, measured here in proportions of sites differing, increases significantly with geographic distance in *S. cowlesi* and *U. ornatus*, showing a pattern of isolation by distance (Mantel tests; **; *p* < 0.01). However, pairwise comparisons (i.e., lava flow nonlava flow) of individuals in all species do not show higher genetic distances than one would expect under an isolation by distance framework, indicating that none of the lava flows are barriers to gene flow

Our PCA analyses showed some genetic variation across populations within each species (Figure 2). *Crotaphytus collaris* and *S. cowlesi* populations showed little structuring by population or habitat, while *U. ornatus* populations appeared to cluster separately. However, PCA does not assess whether patterns of genetic variation are better explained by IBD or IBE. Our ngsDist and Mantel analysis indicated that genetic differentiation within population trios often followed patterns of IBD, but not IBE (Table 1). Genetic distance increased significantly with geographic distance for PALF‐*Sceloporus* and AALF‐*Urosaurus* (*r* = 0.620, *p* = 0.001; *r* = 0.629, *p* = 0.001, respectively) as one would expect under IBD (Table 1; Figure 3). *Crotaphytus* was the only species in which genetic distance was not correlated with geographic distance.

**Table 1:** Mantel and partial Mantel tests in population trios

	Isolation by distance (Mantel)		Isolation by environment (partial Mantel)	
	*r*	*p*	*r*	*p*
PALF‐*Crotaphyuts*	0.158	0.071	−0.189	0.987
CZLF‐*Crotaphytus*	0.136	0.137	0.423	0.003
PALF‐*Sceloporus*	0.453	0.003	0.166	0.078
AALF‐*Urosaurus*	0.629	0.001	−0.419	0.999

Note

Tests for isolation by distance (IBD) examine whether genetic differentiation increases with geographic distance, while tests for isolation by environment (IBE) examine whether genetic differentiation is greater across habitats (lava to nonlava) than within habitats (nonlava to nonlava), while controlling for the effect of geographic distance. While *Sceloporus cowlesi* and *U. ornatus* populations show significant IBD, only CZLF‐*Crotaphytus* shows significant IBE.

Abbreviation: AALF: Aden Afton Lava Flow; CZLF: Carrizozo Lava Flow; PALF: Pedro Armendariz Lava Flow.

Partial Mantel tests confirmed that genetic differentiation could not be explained by environmental differences, even when correcting for geographic distances (Table 1). Only CZLF‐*Crotaphytus* showed an increase in genetic distance with environmental distance when controlling for geographic distance (IBE; *r* = 0.423, *p* = 0.003). All of our population trios did, however, show signals of spatial autocorrelation (all *p* < 0.001 for tests of Moran's *I*). Given that partial Mantel tests have high false‐positive rates with spatially autocorrelated datasets, but not inflated false‐negative rates, our negative results seem robust. The positive association between genetic distance and environmental distance in the CZLF‐*Crotaphytus* population trio may be a false‐positive (Bradburd et al., 2013; Guillot & Rousset, 2013).

Demographic modeling revealed that migration between lava flow and nonlava flow populations occurs often, and regardless of phenotypic differentiation. *F*_ST_ values indicate that levels of genome‐wide differentiation are roughly comparable regardless of whether comparing within or between habitat types (Table 2). Demographic analyses using *∂a∂i* revealed that migration onto and off of the lava flows is likely relatively common. All species showed evidence of migration between at least one lava flow and nonlava flow population (Table S2). There was no consistent pattern observed in terms of patterns of migration and lava flow age or background color homogeneity. In *U. ornatus*, the best model predicted asymmetric migration with stronger migration from nonlava flow populations to the lava flow populations. In *C. collaris*, the best model indicated symmetrical migration between the PALF‐*Crotaphytus* and one nonlava flow population. For the other lava flow in *C. collaris*, the best model suggested relatively high migration between CZLF‐*Crotaphytus* and one nonlava flow population (OS), but AIC values were too close (>10) to determine whether migration was symmetric or asymmetric. Finally, in *S. cowlesi*, the best model predicted migration between PALF‐*Sceloporus* and one nonlava flow population (PALF – MC) but could not distinguish whether migration is symmetric or asymmetric. Thus, regardless of levels of phenotypic divergence between lava flow and nonlava flow populations, migration is ongoing across populations within species.

**Table 2:** *F*_ST_ values for population trios

a) PALF‐*Crotaphytus*		
	**PALF**	BG
BG	**0.066**	
EB	**0.063**	0.088
b) CZLF‐*Crotaphytus*		
	**CZLF**	CP
CP	**0.068**	
OS	**0.065**	0.053
c) PALF‐*Sceloporus*		
	**PALF**	BD
BD	**0.113**	
MC	**0.104**	0.082
d) AALF‐*Urosaurus*		
	**AALF**	CR
CR	**0.197**	
OM	**0.371**	0.286

Note

Lava flow populations and comparisons that include the lava flow populations are highlighted in bold. Comparisons between lava flow and nonlava flow populations show similar levels of differentiation as comparisons between nonlava flow and nonlava flow populations.

Abbreviation: AALF: Aden Afton Lava Flow; BG: Big Gyp Mountain; CP: Carrizozo Private Land Partnership;CR: Corralitos Ranch Road; CZLF: Carrizozo Lava Flow; EB: Elephant Butte; MC: Mesa Camp; OM: Organ Mountains; OS: Oscura Mountains; PALF: Pedro Armendariz Lava Flow.

## 4 DISCUSSION

We demonstrated that genome‐wide differentiation does not accompany local adaptation in three species of lava flow lizards in the Chihuahua Desert. Consistent with previous literature (Lewis, 1949, 1951), we found that *C. collaris* and *S. cowlesi* from the CZLF (~5,200 years old) and *U. ornatus* from the Aden Afton Lava Flow (~106,000 years old) were melanistic, indicating strong phenotypic divergence from nonlava flow populations. Despite what was reported in earlier literature (Best et al., 1983), *C. collaris* and *S. cowlesi* from the PALF (~760,000 years old) were not significantly darker than surrounding nonlava flow populations. The phenotypic patterns themselves are intriguing: the only lava flow lizards that were not melanistic were those from the oldest lava flow, where the substrate is less homogenously dark. However, our most important finding was that, irrespective of phenotypic differentiation, the lava populations typically did not exhibit more divergence from nonlava conspecifics than expected based on geography. Most comparisons also revealed that migration occurs between lava flow and nonlava flow populations. Thus, we find some evidence of local adaptation, but little evidence of the accompanying genetic isolation often reported in the IBE and IBA literature.

In our replicated comparisons, we observed a lack of strong environmentally driven genome‐wide differentiation, regardless of the geological age of the lava flow. The three lava flows differ in age, substrate color, and habitat uniformity, but populations typically showed patterns more consistent with IBD rather than IBE. If there was habitat‐specific genetic differentiation, we would expect greater genetic divergence between lava and nonlava populations (compared with background nonlava to nonlava comparisons). In most cases, overall divergence between lava and nonlava populations was low (*F*_ST_ < 0.15; Hartle & Clark, 1997; Frankham, Ballou, & Briscoe, 2002). Even in the one species with greater divergence among populations (*U. ornatus*), genetic differentiation between lava and nonlava populations was comparable to background differentiation. Moreover, we did not uncover a signal of genetic divergence accumulating or decaying with lava flow age. For example, in *Crotaphytus*, *F*_ST_ between lava and nonlava populations was comparably low regardless of whether the lava flow was 5,200 or 760,000 years old. Using partial Mantel tests, only one comparison (CZLF‐*C. collaris*) showed significant genetic differentiation due to the environment, and even in this comparison, the magnitude of genetic differentiation between habitats was small (Figure 3 and Table 2). Thus, when we detected any genetic differentiation, it appears, as found in some other recent studies (Aguillon et al., 2017; Heath, Schrey, Ashton, Mushinsky, & McCoy, 2012; Wang, Glor, & Losos, 2013), that geographic factors such as distance are more important in shaping genetic patterns than environmental factors or local adaptation (Figure 3).

One explanation for lack of genome‐wide differentiation is ongoing migration between lava flow and nonlava flow populations. Shallow divergence times and ongoing migration in geologically novel habitats (like recently formed lava flows) can make it difficult to distinguish between demographic scenarios, even with thousands of loci (Pinho & Hey, 2010). Yet, for three of the four population trios tested, we were able to detect recent migration between at least one lava flow and nonlava flow population. Additionally, in the one population trio where we could not explicitly calculate levels of migration, CZLF‐*Sceloporus*, previous work corroborates high levels of gene flow between the lava flow and nonlava flow populations (Rosenblum et al., 2007). There are myriad examples of adaptation despite ongoing gene flow in the literature (e.g., Sambatti & Rice, 2006; Dionne, Caron, Dodson, & Bernatchez, 2008; Andrew, Ostevik, Ebert, & Rieseberg, 2012), and selection–migration balance may be a common reason why locally adapted populations do not show genome‐wide differentiation.

There are alternative reasons why lava flow lizards may not show genome‐wide differentiation with phenotypic differentiation. Melanism has a genetic basis in many animal species (van't Hof, 2011; Kingsley, Manceau, Wiley, & Hoekstra, 2009; Nachman, Hoekstra, & D'Agostino, 2003; Schneider et al., 2012), but it is possible that phenotypic plasticity contributes to melanism in these lava flow lizards (Corl et al., 2018; Luke, 1989). Previously, we demonstrated that physiological color change plays a role in the coloration of *C. collaris* at PALF (Krohn & Rosenblum, 2016). However, we have no evidence for developmental plasticity, and other studies demonstrate that melanism is heritable in other lava flow lizards (Corl et al., 2012; Micheletti et al., 2012; Rosenblum, 2005). If melanism has a genetic basis in the species studied here, it is likely few genes control coloration, such that relatively little of the genome is under divergent selection (Gagnaire, Pavey, Normandeau, & Bernatchez, 2013; Nachman et al., 2003; Nosil, Funk, & Ortiz‐Barrientos, 2009; Rosenblum, Rompler, Schoneberg, & Hoekstra, 2010). Additionally, if adaptive loci are not linked to traits involved in reproductive isolation, genome‐wide differentiation is less likely to occur (Feder et al., 2012; MacNair & Christie, 1983; Presgraves, 2013; Slatkin, 1987). Interestingly, a previous study in this system found no preference for local mates in either lava flow or nonlava *S. cowlesi *(Hardwick et al., 2013). Thus, there is no evidence for preferential mate choice or reproductive isolation across lava and nonlava *S. cowlesi* populations, despite phenotypic differentiation. Without a link between the genetic mechanisms of melanism and reproductive isolation, gene flow could easily persist even with selection for background‐matched lizards.

Our replicated results show that regardless of the species studied or the age of the lava flow, locally adapted populations show ongoing gene flow and no more genome‐wide differentiation than expected by geographic distance. Clearly, adaptation does not always lead to genome‐wide divergence, or speciation. Given the similarity of our results across populations, it is possible that selection–migration balance without genome‐wide differentiation may be very common (Hendry, 2009). However, given the claim that incipient ecological speciation is widespread, that IBE is common, and that studies that fail to find large effects of the environment on genetic differentiation are seldom published (Feder et al., 2012; Hendry, 2009; Sexton et al., 2014; Shafer & Wolf, 2013), it is important to also highlight natural systems where local adaptation occurs without genome‐wide differentiation.

## CONFLICT OF INTERESTS

None declared.

## AUTHOR CONTRIBUTIONS

A.R. Krohn designed the experiment, conducted the field and laboratory work, analyzed the data, and wrote the manuscript. E.T. Diepeveen contributed to fieldwork and laboratory work. K. Bi contributed to data analysis. E.B. Rosenblum contributed to the experimental design and manuscript preparation.

## Data Availability

Raw sequence data are archived in the Short Read Archive (BioProject Accession PRJNA417310).
